# Can consumers learn to ask three questions to improve shared decision making? A feasibility study of the ASK (AskShareKnow) Patient–Clinician Communication Model^®^ intervention in a primary health‐care setting

**DOI:** 10.1111/hex.12409

**Published:** 2015-09-14

**Authors:** Heather L Shepherd, Alexandra Barratt, Anna Jones, Deborah Bateson, Karen Carey, Lyndal J Trevena, Kevin McGeechan, Chris B Del Mar, Phyllis N Butow, Ronald M Epstein, Vikki Entwistle, Edith Weisberg

**Affiliations:** ^1^ Centre for Medical Psychology and Evidence‐based Decision‐makingSydney School of Public HealthThe University of SydneySydneyNSWAustralia; ^2^ Family Planning NSWSydneyNSWAustralia; ^3^ Discipline of Obstetrics, Gynaecology and NeonatologyThe University of SydneySydneyNSWAustralia; ^4^ Health Consumers CouncilPerthWAAustralia; ^5^ Centre for Research in Evidence Based PracticeBond UniversityRobinaQLDAustralia; ^6^ Center for Communication and Disparities ResearchUniversity of Rochester Medical CenterRochesterNYUSA; ^7^ Health Services Research UnitUniversity of AberdeenAberdeenUK

**Keywords:** communication, consumer, patient empowerment, patient involvement, shared decision making

## Abstract

**Objective:**

To test the feasibility and assess the uptake and acceptability of implementing a consumer questions programme, AskShareKnow, to encourage consumers to use the questions ‘1. What are my options; 2. What are the possible benefits and harms of those options; 3. How likely are each of those benefits and harms to happen to me?’ These three questions have previously shown important effects in improving the quality of information provided during consultations and in facilitating patient involvement.

**Methods:**

This single‐arm intervention study invited participants attending a reproductive and sexual health‐care clinic to view a 4‐min video‐clip in the waiting room. Participants completed three questionnaires: (T1) prior to viewing the intervention; (T2) immediately after their consultation; and (T3) two weeks later.

**Results:**

A total of 121 (78%) participants viewed the video‐clip before their consultation. Eighty‐four (69%) participants asked one or more questions, and 35 (29%) participants asked all three questions. For those making a decision, 55 (87%) participants asked one or more questions, while 27 (43%) participants asked all three questions. Eighty‐seven (72%) participants recommended the questions. After two weeks, 47 (49%) of the participants recalled the questions.

**Conclusions:**

Enabling patients to view a short video‐clip before an appointment to improve information and involvement in health‐care consultations is feasible and led to a high uptake of question asking in consultations.

**Practice Implications:**

This AskShareKnow programme is a simple and feasible method of training patients to use a brief consumer‐targeted intervention that has previously shown important effects in improving the quality of information provided during consultations and in facilitating patient involvement and use of evidence‐based questions.

## Introduction

Shared decision making is considered key to supporting patients to understand treatment and possible options, express values and preferences to make good treatment decisions and better manage their health.^1^ However, the greatest challenge to the widespread implementation of shared decision making remains encouraging clinicians to adopt it. One avenue is to encourage consumers to initiate the process, which can be achieved by them asking a few key questions. Consumer health organizations (e.g. the Patient First project), and publications for consumers (e.g. Smart Health Choices) use this approach.^2^, ^3^ Accordingly, this approach has been developed in the form of Question Prompt Lists (QPLs), which improve patient involvement in decisions and enhance patient knowledge and realistic expectations about outcomes.^4^ Unfortunately, QPLs have been developed only for specific clinical contexts, such as cancer treatment choices, and are often long and challenging to distribute to patients in time to be relevant to decision making.

To address these challenges, we previously conducted a crossover controlled trial to test whether three generic questions could facilitate communication about treatment options independently of the clinical context.^5^ The three questions, which aim to elicit the minimum information needed for decision making under conditions of uncertainty and to help organize the information that physicians give patients, are shown in Box 1.

Box 1 **The AskShareKnow Questions**

1. What are my options?
2. What are the possible benefits and harms of those options?
3. How likely are each of those benefits and harms to happen to me? Including ‘What will happen if I do nothing?’

In this prior study involving trained actors as unannounced standardized patients, we showed that these questions significantly increased facilitation of patient involvement in the consultations, as well increasing information given by physicians about options and their associated benefits and harms.^5^ Encouraged by these positive findings, we developed a programme to train and support consumers to ask these three questions which involved them viewing a 4‐min video‐clip in the waiting room before an appointment. This paper reports a study that aimed to test the feasibility of implementing this programme in routine care in a primary care setting, and to assess uptake and acceptability.^6^

## Methods

### Study design

This was a single‐arm intervention study to test the feasibility of the intervention, for which we developed a consumer questions training and support programme, now called AskShareKnow (ASK Patient–Clinician Communication Model^®^). The intervention included three components: a 4‐min video‐clip that participants were shown in the waiting room (a 1 and 9‐min versions were also available on the website); a pamphlet which incorporated a consultation summary sheet and website information (www.askshareknow.com.au); and a refrigerator magnet as a reminder for future use.

### Study Setting and Participants

The study was conducted at a metropolitan family planning clinic (part of a statewide non‐government organization) providing reproductive and sexual health‐care services, primarily to women. Patients >18 years of age, and with English fluency, attending appointments at the clinic were invited to participate in the study. Those with infants were excluded for logistical reasons.

Family planning consultations are ideal for testing shared decision‐making interventions because they encompass a very wide range of treatment options (for example, which type of contraception to choose, or whether or not to use hormone therapy at the time of menopause) with near equipoise of benefits and harms, and are therefore very sensitive to consumer preference. Furthermore, women – the overwhelming consumers in this clinic – are usually the primary decision‐maker of health matters in society and have influence in over 80% of health‐care decisions.^7^, ^8^, ^9^, ^10^, ^11^, ^12^

Recruitment took place between July and December 2012. The study was approved by the Human Research Ethics Committees of Family Planning NSW and the University of Sydney. Detailed study information was provided to participants and written consent obtained. The trial was registered with the Australian New Zealand Clinical Trials Registry no. ACTRN12610001055099.

### Study procedures

After providing consent, participants were provided with the three components of AskShareKnow described above, and asked to complete a questionnaire prior to their consultation (T1). They were then given a media tablet with headphones to watch the 4‐min AskShareKnow video‐clip (but not otherwise invited to ask the three questions featured). To avoid disrupting clinic flow, appointment scheduling took precedence over recruitment, so not all who were recruited were exposed to all the intervention. Those who watched the video‐clip prior to their consultation were invited to complete a second questionnaire immediately after the consultation (T2), and then a third questionnaire at 2 weeks (T3) (posted or emailed with a link to complete the survey online, according to preference). Questionnaire items included quantitative and qualitative responses. Quantitative responses were analysed using descriptive statistics. Participants were also invited to take part in a semi‐structured telephone interview at four to six weeks to obtain more detailed information about their experience and views of the AskShareKnow programme. Telephone Interviews were audio‐recorded transcribed verbatim and analysed using thematic analysis.

### Study outcomes

The primary outcome was the use of the three questions in the health‐care consultation based on self‐report by the participants. Other outcomes focussed on key demand, acceptability and implementation aspects of AskShareKnow^6^: access to the ASK online information (demand); use of consultation summary sheet (demand); attitudes to the ASK Patient–Clinician Communication Model (acceptability); question recall (implementation); number of times AskShareKnow video‐clip was watched (Implementation); time to view AskShareKnow website and pamphlet (implementation); understanding of AskShareKnow video‐clip (implementation).

Data were collected at three time points. The baseline questionnaire (T1) included demographic information, decision making and information preferences^13^, ^14^; the second questionnaire (T2) included study‐specific information about the reason for the consultation (whether there was a health‐care decision to make, comments on the AskShareKnow materials, and post‐consultation decision making and information preferences); while the third questionnaire (T3) collected information about recall of the intervention (whether they had watched the video‐clip again, had read the information available on the AskShareKnow website, and if they had recommended the questions to others).

## Results

### Participants

**Figure 1:**
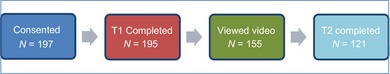
Recruitment flow.

A total of 197 participants consented to the study; however, participants who had not completed viewing the video‐clip before being called into their consultation (see Methods) were excluded. (See Fig. 1) Of the 155 participants recruited into the study who viewed the video‐clip prior to their consultation, 121 (78%) completed the second questionnaire (T2). The 121 were considered the final sample and included in the analyses.

Six study participants and four clinicians participated in semi‐structured telephone interviews exploring their experience of asking, or being asked, the questions.

Thirty‐three women (27%) were born outside Australia, 73 (60%) university educated, 70 (58%) aged under 40 years (Table 1). Information preferences were high with 104 (87%) wanting as much information as possible, with over a third (38%) stating a preference for decisions to be made together on an equal basis, and 55 (45%) wanting to play a lead role in decision making (Table 1). Reasons for visits were reported by 63 participants: 32 (51%) related to contraceptive options with the remainder seeking advice regarding pregnancy, menstruation, sexually transmissible infections and general sexual and reproductive health.

**Table 1:** Baseline characteristics of study sample (*n* = 121)

	*N*	%
Age		
20–39 years	70	58
40–59 years	43	36
>60 years	7	6
Marital status		
Single/never Married	42	35
Married/de facto	68	56
Separated/divorced	11	9
Born in Australia	88	73
Education achievement		
Year 10 (16 years)	7	6
Year 12/HSC (18 years)	11	9
TAFE (Technical College)	30	25
University	73	60
Involvement preferences		
The doctor should make the decisions using all that's known about the treatments	2	2
The doctor should make the decisions but strongly consider my needs and priorities	18	15
The doctor and I should make the decisions together on an equal basis	46	38
I should make the decisions, but strongly consider the doctor's opinion	49	40
I should make the decisions using all I know or learn about the treatments	6	5
Information preferences		
Prefer as many details as possible	81	68
I want only information needed to care for myself properly	15	13
I want additional information only if it is good news	0	0
I want as much information as possible, good and bad	104	87

Demographic information of those who consented but did not have sufficient time prior to their consultation to view the video‐clip was similar to the final sample included in the analysis.

### Demand

**Figure 2:**
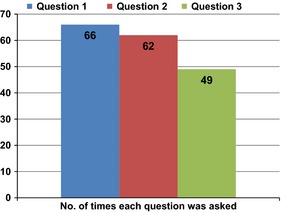
Number of times each question asked.

Eighty‐four participants (69%) reported asking at least one of the questions during their consultation, with 35 (29%) asking all three Questions (Table 2). Participants were asked whether they had a health‐care decision to make during their consultation, and this information was compared to whether they asked one or more of the questions (Table 2). Of the 63 participants who reported making a decision, 55 (87%) asked at least one of the Questions, with 27 (43%) asking all three Questions. In comparison, of participants who had no decision to make (*n* = 58), 29 (50%) asked at least one of the Questions and 8 (14%) asking all three Questions. Of the three Questions, 1 and 2 were asked by 66 and 62 participants, respectively, while Question 3 was asked by 49 (Fig. 2).

**Table 2:** ASK questions asked in consultation

No. of Questions asked		Was a decision made?	
	*n* (%)	Yes	No
		*n* (%)	*n* (%)
≥ 1 question	84 (69)		
3 questions	35 (29)	27 (43)	8 (14)
2 questions	25 (21)	18 (28)	7 (12)
1 question only	24 (20)	10 (16)	14 (24)
None	37 (31)	8 (13)	29 (50)

The other components of the ASK Patient–Clinician Communication model were less utilized by participants in the study. Thirty‐eight (31%) had time to view the AskShareKnow website prior to their consultation, and 58 (48%) reported reading the pamphlet prior to their consultation, with 19 (16%) reporting using the consultation summary sheet during the consultation.

Several of the participants who were interviewed reported that the questions were similar to those they would normally ask, although commented the questions provided additional structure (Box 2i) .

Box 2 **Sample comments from the follow up interviews**

**Table:** 

i.	I think it just, as I said I didn't do it in the exact order, I used kind of my own version of it but it reminded me while I was in the waiting room I just kind of remembered that that would be useful. It is a good process to have in your head. So not totally different, I am sure that I would have got a lot of the information had I not asked them through conversation but it was definitely good for me to remember to kind of focus in on those points as well. P175
ii.	Yes, look, I think the questions could be useful but possibly in a consultation with a GP or specialist when there might be more unknowns or some bigger decisions to be made. HP04
iii.	It's funny because going to the doctor isn't something that you are ever trained to do [..] having someone to go “ok, well you can ask these questions”, so you go in and you feel like you have more of a role in the whole thing. [..] And once you've been a few times then you can say, “oh well, I asked it this way last time and I got this sort of information, but now I can adapt that too, from my experience, to the rest of life” so yeah, it is nice to have someone say here's how you can go about getting more information. P129
iv.	No it helped! It helped a lot. I definitely got more information. It was very positive. It made me realise that we don't ask questions enough and we don't ask about side effects [..]The information I got back when I asked the questions [..] I wouldn't have found that out if I hadn't asked. So it just made me want to question things more, to get clearer information about side effects and general questions. The doctor was very open to talking about things, and to give me the information I wanted [..] I walked out feeling really good. P85
v.	it is always good to see a visual cue, to remind you of what's going on [..] I thought I understood the first time but it helped to sort of clarify it in my head [..] having that visual aide to help explain things P129
P – Patient	
HP – Health professional

The clinicians (*n* = 4) interviewed commented that the questions covered information that would generally be covered during a consultation, but all stated it was useful for participants to be provided with the questions in the waiting room, although one did raise query whether they were necessary in all consultation types (Box 2ii).

### Acceptability

After the consultation, most participants, 87 (72%), reported they would definitely recommend the questions. Two weeks later, 47 (83%) of those completing the final questionnaire would use the questions again. Each of the participants (*n* = 6) who were interviewed stated they would ask the questions again, although not necessarily verbatim (Box 2iii).

Most participants who reported making a decision during the consultation rated the questions as very helpful 33 (53%), or somewhat helpful 19 (31%). This reflected comments made by participants who were interviewed (Box 2iv).

### Implementation

Participants were asked whether they recalled the three questions two weeks after their consultation (T3). Of the 95 participants who responded, 47 (49%) could; of 62 participants who provided details of the questions, 29 (47%) were able to recall all three questions accurately (Table 3). Six participants (10%) recalled the AskShareKnow slogan rather than questions. Participant self‐rated understanding of the AskShareKnow video‐clip was high, with the majority of participants (88%) reporting that the information provided on the video‐clip was clear; 119 (98%) felt comfortable watching the video‐clip in the waiting room. Four participants said they had watched the video‐clip a second time and 62 (65%) reported they had read the information on the website. Suggestions from participants (either within the interview, or questionnaire) included reducing the length of the video‐clip by making the information more succinct; having a variety of medical situations; being less gender specific; and adding the questions as text during the scenarios. Overall, the response to the video‐clip was positive. Five of the six participants who were interviewed reported that the video‐clip had helped them understand why the three questions were being recommended and suggested that continued exposure to the questions could aid people's recall. Two of the six added that it was useful to see the questions being asked (Box 2v).

**Table 3:** Question recall at 2 weeks

Accurate recall	*N* (%)
Question 1 What are my options?	51 (82)
Question 2 What are the possible benefits and harms of those options?	38 (61)
Question 3 How likely are each of those benefits and harms to happen to me?	30 (48)
All 3 questions	29 (47)
Would ask the questions again	47 (83)

The clinicians interviewed did not identify any logistical issues during the recruitment phase of the study, either in terms of the study intervention causing a delay to consultations or adding length to the consultation due to clients wishing to discuss the intervention.

## Discussion

This study demonstrated that the brief AskShareKnow intervention was successful in prompting participants to ask the three questions in their subsequent consultation, was acceptable to patients and feasible in a busy primary care setting.

The three questions at the basis of the AskShareKnow programme were designed to assist patients to make informed, as well as evidence‐based, decisions.^5^ These results show that participants who reported making a decision in their consultation were more likely to report asking all three questions. This suggests that participants facing decisions thought that asking the questions could be useful in obtaining information they needed to reach a decision with the clinician. In our first study, we found that distinguishing between answers that patients receive from Question 2 (the possible outcomes) and Question 3 (their probabilities) was difficult to explain to the trained actors whom we used as consumers. However, the results from this study demonstrate that participants used the questions appropriately.

This study adds valuable information about implementation to the many studies that report positive effects of interventions to support information provision, and question asking, to improve participation in decision making. A meta‐analysis of interventions to promote question asking using question‐listing interventions that included 33 randomized controlled trials and over 8000 patients found an increase in question asking.^15^ Studies that have investigated implementation include the Situation–Choices–Objectives–People–Evaluation–Decisions (SCOPED) question‐listing intervention^16^ and report that it is feasible, and was implemented in their pilot study with high fidelity and effectiveness determined by low levels of distress and anxiety and increased question self‐efficacy. This intervention, however, relies on the availability of trained coaches within health‐care settings, and although this approach is valued by patients, there are concerns about its sustainability. Another implementation study investigating a use of QPL for patients consulting a medical or radiation oncologist reported that 41% patients said it helped them ask their clinician more questions than they would have ordinarily asked,^17^ with the QPLs being handed out by nursing, medical or reception staff within the participating cancer centres.

The simplicity of the AskShareKnow programme intervention and the study findings suggests that a short video‐clip, website and pamphlet may be sufficient to promote patient engagement and facilitate shared decision making. The persistence of the message is unknown, but of course offering resources like this in waiting rooms to patients could be repeated for each consultation as a reminder.

Between October 2010 and August 2013, the UK Health Foundation through its MAGIC (MAking Good decisions In Collaboration http://www.health.org.uk/areas-of-work/programmes/shared-decision-making/) programme used the three questions as one of its tools to promote SDM. Evaluation of this component was limited to the specific marketing campaign, and although this was positive (patients and clinicians liked it), the effectiveness of the campaign remains uncertain.^18^, ^19^ This adds to the challenge for SDM researchers in identifying measurable outcomes that demonstrate effectiveness of this and similar interventions.

Limitations of this study include relying on patient self‐reports that questions were asked; the lack of recording consultations and the generalizability of study outcomes (given the relatively high education level of the participants, a setting in which service users recognize decisions are being made in a large proportion of consultations and that those decisions feature options about which people might reasonably have different opinions). While recall of the questions was high at 2 weeks, and participants reported that the questions were useful, and that they intended to recommend them to others, whether or not participants would repeat their use is unknown. Additionally, our exclusion of participants who did not have time to complete watching the 4‐min video‐clip reduced our sample; however, this was caused by the requirement in the research setting to gain consent and complete baseline questionnaires in addition to viewing the video‐clip.

## Conclusion

The brief consumer questions training and support programme, AskShareKnow (ASK Patient–Clinician Communication Model^®^) enabled consumers to ask the listed questions in health‐care consultations. Patients were able to view a short video‐clip to promote question asking immediately before their consultation, and most went on to ask at least one of the questions in that consultation.

### Practice implications and future research

This brief intervention of three generic questions relating to health‐care decisions has shown important effects in improving the quality of information provided during clinical consultations and in facilitating patient involvement. It demonstrates the utility of the intervention through the ability of patients to use the questions following a brief waiting room video‐clip demonstration. While further evaluation to determine generalizability of study findings to other settings is needed and could add to the evidence by investigating the longer term impact of a larger campaign, should health systems proceed with implementing the AskShareKnow intervention at an institutional, regional or even national level as a simple way for patients and clinicians to share decisions in practice?

## Contributors

HS conceived the study. HS, AB, DB, KC, LT, KM, CDM, PB, RM, VE and EW designed the study and obtained funding. AJ coordinated the running of the study and the data collection. KM carried out the statistical analysis. HS wrote the first draft of the manuscript. All authors contributed to the interpretation of the analysis and the writing of the manuscript. All authors are guarantors.

## Funding

This work was supported by Grant #0175‐1 from the Informed Medical Decisions Foundation. Dr. Shepherd received salary support from National Health and Medical Research Council Public Health Training Fellowship ID 568962. Dr. Shepherd had full access to all of the data in the study and takes responsibility for the integrity of the data and the accuracy of the data analysis.

## References

[1] Barry MJ , Edgman‐Levitan S . Shared decision making — the pinnacle of patient‐centered care. New England Journal of Medicine, 2012; 366: 780–781. 22375967 10.1056/NEJMp1109283

[2] Irwig L , Irwig J , Trevena L , Sweet M . Smart Health Choices, Rev. and Updated Ed edition ed. London: Hammersmith Press, 2008: 256. 22013602

[3] Evans I , Thornton H , Chalmers I . Testing Treatments: Better Research for Better Healthcare, 1st reprint edition ed. London: Pinter & Martin; 2010: 128. 22171402

[4] Trevena LJ , Davey HM , Barratt A , Butow P , Caldwell P . A systematic review on communicating with patients about evidence. J Eval Clin Pract, 2006; 12: 13–23. 16422776 10.1111/j.1365-2753.2005.00596.x

[5] Shepherd HL , Barratt A , Trevena LJ *et al* Three questions that patients can ask to improve the quality of information physicians give about treatment options: a cross‐over trial. Patient Education and Counseling, 2011; 84: 379–385. 21831558 10.1016/j.pec.2011.07.022

[6] Bowen DJ , Kreuter M , Spring B *et al* How we design feasibility studies. American Journal of Preventive Medicine, 2009; 36: 452–457. 19362699 10.1016/j.amepre.2009.02.002 PMC2859314

[7] LaCoursiere SP . A theory of online social support. Advances in Nursing Science, 2001; 24: 60–77. 11554534 10.1097/00012272-200109000-00008

[8] James G . Winning in The Women's Health Care Marketplace: A Comprehensive Plan for Health Care Strategists. San Francisco: Jossey Bass, 1997.

[9] Nussbaum R . Studies of women's health care: selected results. Perm J, 2000; 4: 62–67.

[10] Pandey SK , Hart JJ , Tiwary S . Women's health and the internet: understanding emerging trends and implications. Social Science and Medicine, 2003; 56: 179–191. 12435560 10.1016/s0277-9536(02)00019-9

[11] Carpenter C , ed. Empowering consumers to be better health care decision‐makers. Intel Internet Health Conference; 1998 October 27; San Francisco, CA.

[12] Fox S , Rainie L . The Online Health Care Revolution: How the Web Helps Americans Take Better Care of Themselves. Washington, DC: The Pew Internet and American Life Project, 2000.

[13] Degner LF , Sloan JA . Decision making during serious illness: what role do patients really want to play? Journal of Clinical Epidemiology, 1992; 45: 941–950. 1432023 10.1016/0895-4356(92)90110-9

[14] Cassileth BR , Zupkis RV , Sutton‐Smith K , March V . Information and participation preferences among cancer patients. Annals of Internal Medicine, 1980; 92: 832–836. 7387025 10.7326/0003-4819-92-6-832

[15] Kinnersley P , Edwards A , Hood K *et al* Interventions before consultations to help patients address their information needs by encouraging question asking: systematic review. British Medical Journal, 2008; 337: a485. 18632672 10.1136/bmj.a485 PMC2500196

[16] Belkora J , Miller M , Crawford B *et al* Evaluation of question‐listing at the Cancer Support Community. Transl Behav Med, 2013; 3: 162–171. 24073167 10.1007/s13142-012-0186-8 PMC3717975

[17] Dimoska A , Butow PN , Lynch J *et al* Implementing patient question‐prompt lists into routine cancer care. Patient Education and Counseling, 2012; 86: 252–258. 21741195 10.1016/j.pec.2011.04.020

[18] Lloyd A , Joseph‐Williams N , Edwards A , Rix A , Elwyn G . Patchy ‘coherence’: using normalization process theory to evaluate a multi‐faceted shared decision making implementation program (MAGIC). Implementation Science, 2013; 8: 1–9. English. 24006959 10.1186/1748-5908-8-102 PMC3848565

[19] King E , Taylor J , Williams R , Vanson T . The MAGIC Programme: Evaluation. London: Office for Public Management, 2013.

